# The effect of anger expression style on cardiovascular responses to lateralized cognitive stressors

**DOI:** 10.1007/s40708-017-0068-4

**Published:** 2017-05-15

**Authors:** David E. Cox, Benjamin B. DeVore, Patti Kelly Harrison, David W. Harrison

**Affiliations:** 1 Virginia Polytechnic Institute and State University, Blacksburg, VA 24061 USA; 2 Florida Gulf Coast University, 10501, FGCU Blvd S., Fort Myers, FL 33965 USA; 3 Department of Psychology, Behavioral Neuroscience Laboratory, Williams Hall, Virginia Tech, Blacksburg, VA 24061-0436 USA

**Keywords:** Hostility, Anger, Cardiovascular response, Brain asymmetry, Laterality

## Abstract

To determine the effects of self-reported anger expression style on cerebrally lateralized physiological responses to neuropsychological stressors, changes in systolic blood pressure and heart rate were examined in response to a verbal fluency task and a figural fluency task among individuals reporting either “anger in” or “anger out” expression styles. Significant group by trial interaction effects was found for systolic blood pressure following administration of verbal fluency [*F*(1,54) = 5.86, *p* < 0.05] and nonverbal fluency stressors [*F*(1,54) = 13.68, *p* < .001]. Similar interactions were seen for systolic heart rate following administration of verbal fluency [*F*(1,54) = 5.86, *p* < .005] and nonverbal fluency stressors [*F*(1,54) = 13.68, *p* < .001]. The corresponding results are discussed in terms of functional cerebral systems and potential implications for physiological models of anger. Given the association between anger and negative physical health outcomes, there is a clear need to better understand the physiological components of anger. The results of this experiment indicate that a repressive “anger in” expression style is associated with deregulation of the right frontal region. This same region has been shown to be intimately involved in cardiovascular recovery, glucose metabolism, and blood pressure regulation.

## Introduction

Elements of the multifaceted emotional construct of anger, such as hostility, have been shown to be associated with increased risk of negative health outcomes such as cardiovascular disease [1–3], cerebrovascular accident [4], and the metabolic syndrome [5, 6]. Not surprisingly, anger represents one of the most frequently investigated emotional constructs over the past decade [7]. Despite the frequency of investigation and the health implications ascribed to anger, the mechanisms by which this emotional construct impacts physiological functions are not fully understood. One mechanistic model offered considers specifically how an individual deals with the experience of anger. This model postulates individuals may process anger through either externalizing or internalizing the feelings. Although there has been substantial debate regarding which of these strategies may lead to negative health outcomes, there have been surprisingly few empirical investigations examining the strategies underlying these two expression styles. The present research examines changes in functional cerebral systems associated with relevant physiological processes among individuals reporting different anger expression styles.

### Defining anger and anger expression

Before any investigation can be conducted in the area of anger, the construct must be clearly defined. Many different definitions have been offered, and the construct has been divided into multiple constructs and factors. For the purposes of the current research, anger was operationally defined as a multidimensional construct with distinct affective, behavioral, and cognitive dimensions that include specific physiological elements, which contribute to both the experience and expression of the emotion [8]. The affective dimension of anger refers to the emotional state, which occurs in response to an immediate stressor and may vary in both intensity and duration [9]. The cognitive dimension of anger, also referred to as hostility in the literature, has most frequently been defined as a cognitive phenomenon of an attitudinal nature that subserves the emotional process, but is not an emotion per se [10]. The behavioral dimension of anger is simply the behavioral response to the subjective experience of anger [11] and may be expressed outwardly or inwardly [9].

Anger expression styles refer to the manner in which an individual expresses her/his emotional experience of anger. Spielberger et al. ([9]; see also [12, 13]) suggested that the tendency to express one’s anger in an outwardly negative manner represented an outward-directed style known as anger out (A.O.). Anger out may involve the use of aggressive actions (e.g., assaultive behavior, destruction of property, or making offensive gestures) and/or aggressive verbal behavior (e.g., insults, offensive/inappropriate language, or shouting). Individuals displaying the A.O. style may choose targets for hostile or aggressive behavior if the target is seen as even remotely related to the cause of their anger, including mere proximity when the outburst occurs [8]. Conversely, the concept of anger in (A.I.) refers to the extent to which individuals suppress anger when they are experiencing this negative emotion [9]. High levels of anger suppression have been found to lead to the angry feelings being suppressed and replaced with guilt, anxiety and depression as the person blames himself for the problems surrounding the anger-provoking situation [14].

Interaction among the dimensions of anger and anger expression style is of particular interest in the current research. According to Spielberger and colleagues [14], individuals with a higher degree of trait-like cognitive anger are more likely to have more frequent state-like experiences of affective anger. Once an individual experiences affective anger, the emotion may be expressed outwardly or repressed. Although any individual who experiences anger may employ an anger expression style, individuals with chronic, state-like anger are more likely to perceive events in a negative manner and experience anger more frequently [10]. Therefore, these individuals will demonstrate persistent and consistent patterns of anger repression or expression and thus be vulnerable to any effects caused by the expression style. For this reason, the current research is particularly interested in the effects of the differing expression styles among those individuals with high levels of trait-like cognitive anger.

### Physiological sequela of anger

There have been numerous investigations into the effects of anger on physiological function (for review, see [3]; see also [13]). The bulk of the literature in this area conceptualizes anger as an emotion-induced physiological stressor, with the same physiological outcomes associated with environmental stressors [10]. The physiological response to anger is consistent with Selye’s [15] stress model, which maintains that the body increases autonomic arousal in response to a stressor. This increase in autonomic arousal may result in increased cardiovascular activity, glucose metabolism, and changes in patterns of cortical arousal [16, 17]. The bulk of this research has been conducted utilizing either cognitive anger (hostility) or affective anger (experience) as the variable of interest (for review, see [18]). Therefore, it is reasonable to suggest that chronic feelings of anger or the subjective experience of anger are associated with increased autonomic arousal.

In terms of anger expression, there have been fewer investigations into the physiological responses to both A.O. and A.I. expression styles. However, there has been some evidence that A.I., as measured by the Anger-In Scale of the State-Trait Anger Expression Inventory (STAXI; [19]), is associated with increased incidence of coronary artery disease [20], increased blood pressure in response to anger provocation [21], and poorer response to therapeutic interventions [22]. Findings such as these suggest that there may be different patterns of physiological responses to anger based on expression style.

### Functional systems theory

The key to the neuropsychological investigation of emotional expression lies in Luria’s [13] functional cerebral systems theory and Kinsbourne’s [23] cerebral space theory. The functional cerebral systems model proposes that there are specific coordinated regions of the brain that are utilized to complete certain tasks. The functional systems model proposes that multiple regions in different parts of the brain may be involved in similar tasks, so while there may be evidence of diffuse activation in a given process (e.g., expressive speech), the activation patterns will be consistent for that particular activity across individuals [24]. The idea of functional cerebral space maintains that tasks (cognitive, motor, or emotional) require utilization of cerebral resources within a given functional system. If the functional systems that underlie completion of each of two tasks are close in proximity, decrements in performing one of the tasks will be especially evident [23, 25, 26].

The functional cerebral systems approach has been utilized in a number of investigations on the effect of hostility and anger on cardiovascular functions. Research in the area of cardiovascular responses to cognitive and emotional stressors has demonstrated some lateralizing results. Demaree and Harrison [27] predicted increased sympathetic arousal (increased systolic blood pressure, diastolic blood pressure, and heart rate) following cold-pressor exposure at the left arm. Results supported this prediction, with both low- and high-hostile males evidencing increases in systolic blood pressure, diastolic blood pressure, and heart rate. Moreover, the high-hostile group was shown to be significantly more reactive to the cold-pressor as evidenced by a significant group by condition interaction for the heart rate data. Thus, significant global sympathetic arousal was not found for the high-hostile group, but evidence of increased sympathetic tone was noted. Rhodes and Harrison [28] presented a similar finding of a significant interaction between group (low- and high-hostile) and exposure to cold-pressor stress at the left arm. Results showed increased cardiovascular reactivity (heart rate) in the high-hostile group in response to the stressor. Low-hostiles displayed decreased heart rate following the cold-pressor, whereas high-hostiles displayed increased heart rate following the stressor.

Exploration of hemispheric control of sympathetic response, particularly as it relates to blood pressure and overall heart rate regulation, indicates general control of sympathetic response by the right hemisphere, with parasympathetic control lateralized to the left hemisphere [13]. Taking into account, right hemispheric dominance for sympathetic response is an important component when looking at the potential for differing anger expression styles. If, as theorized, taxation of right frontal lobe resources allows for an unbridling of posteriorly located neural regions, it is presumed that tasks which are directed toward the right hemisphere would result in greater activation of the sympathetic response. Similarly, if anger suppression via right frontal lobe control exhausts right frontal lobe resources, consequent release of sympathetic control is expected to result in increased variability within heart rate and blood pressure.

Considering the various health and psychological impacts associated with anger expression, the current research investigated the effects of self-reported anger expression style on cerebrally lateralized physiological responses to a neuropsychological stressor. Specifically, this research examined changes in systole and grip strength in response to a verbal fluency task and a figural fluency task. Previous research has demonstrated that the verbal fluency and figural fluency tasks are appropriate for eliciting performance-related lateralized activation [29], as verbal fluency has been shown to be a stressor for the left anterior region, and design fluency has the same effect on the right anterior region [29, 30]. Based on the preceding review, we predicted that although both groups would demonstrate increased systolic blood pressure and heart rate in response to a nonverbal fluency task, individuals reporting a repressive A.I. style would show significantly greater increases compared to those reporting an expressive A.O. style. Conversely, we predicted that individuals reporting an expressive A.O. style would show increases in systolic blood pressure and heart rate in response to a verbal fluency task, while individuals reporting a repressive A.I. style would show decreased heart rate and blood pressure in response to a verbal fluency measure.

## Methods

### Participants

Participants were recruited from the undergraduate psychology population. They completed an online pre-screening that included an Informed Consent Form; a Medical History Questionnaire; the Coren, Porac, and Duncan Laterality Questionnaire [31]; the Cook-Medley Hostility Scale [32]; and the State-Trait Anger Expression Inventory [8]. In order to control for potential laterality confounds and overall cognitive deficits associated with substance or structural influence, participants reporting left-hand dominance, a history of brain-related insult (e.g., stroke, seizure, and traumatic brain injury.), use of psychotropic medications or significant physical or mental health difficulties that would prohibit or limit their participation were excluded. Additionally, to ensure appropriate levels of trait-like cognitive anger, participants had to score 29 or above on the Cook-Medley Hostility Scale. To ensure appropriate expression of the different anger styles, clinical norms for the State-Trait Anger Expression Inventory (>70th percentile) were used as cutoffs for inclusion in the experiment. Participants who had been included in other studies within the laboratory and had already been shown to be in the high-hostile range were referred to the online screening. Individuals participating in this initial screening were awarded one course credit point for their participation.

Of the 377 students completing the online screening, 62 met criteria for inclusion in the experimental phase and were scheduled for follow-up testing. Among those not included in the experimental phase, most were excluded for not meeting scoring criteria on the Cook-Medley Hostility Scale (*n* = 256) or the State-Trait Anger Expression Inventory (*n* = 97) with others (*n* = 5) excluded for medical or psychiatric conditions (*n* = 5). As the experiment advertisement indicated that right-handed men were being recruited, few individuals were excluded on the basis of handedness (*n* = 3) or gender (*n* = 2). To ensure test–retest reliability, initially accepted participants were administered the screening measures a second time in the laboratory. Further exclusion criteria included participants who scored differently on the screening measures at the second administration. Specifically, individuals who scored in the upper one-third of scores on the CMHI during the online screening, but then scored significantly lower at the follow-up assessment (*n* = 9). Finally, a small set of individuals reporting a primarily A.I. expression style (*n* = 5) were randomly excluded to ensure equal anger expression group sizes. The final analysis included 56 high-hostile men between the ages of 18 and 24 years (*M* = 19.50, SD = 1.50).

## Materials

### Questionnaires

General medical and psychiatric health was assessed using a brief inventory designed for use in neuropsychological research [1, 32]. The Coren–Porac–Duncan Laterality Test [31] was utilized to determine laterality. Cognitive anger was assessed using the Cook-Medley Hostility Inventory (CMHI; [32]), which consists of 50 dichotomous “true/false” items broken into six categories (hostile attributions, cynicism, hostile affect, aggressive responding, social avoidance, and other). The State-Trait Anger Expression Inventory (STAXI; [19]) is a 57-item, 4-point scale inventory, consisting of subscales that measure anger intensity and overall angry feelings. The STAXI has been normed for individuals 16–63 years of age and takes approximately 5–10 min to administer. Elevated *T* scores have been associated with specific anger expression styles. It was used to assess anger expression style since it is purported to measure transient (state) anger expression (anger out) and anger inhibition (anger in).

### Neuropsychological measures

The Controlled Oral Word Association Test (COWAT [33]) functions as a measure of verbal fluency by asking participants to spontaneously name words beginning with an identified specific letter (“*F*” for example) and takes approximately 5–10 min to administer. This instrument has been demonstrated to differentially stress left cerebral systems or circuits processing logical linguistic or propositional speech [34]. The Ruff Figural Fluency Test (RFFT) [35] consists of five separate parts and takes approximately 5 min to administer. It has been shown to provide information regarding nonverbal capacity for fluid and divergent thinking. This instrument has been further demonstrated to differentially stress right cerebral systems or circuits processing figural fluency or design drawing [29].

### Blood pressure and heart rate

The Norelco Healthcare Electronic Digital Blood Pressure/Pulse Meter (Model HC3030; Norelco Health Group, Inc; New York) was used to provide an oscillometric measure of systolic blood pressure and heart rate. This instrument has been shown to demonstrate adequate accuracy and reliability in empirical comparison trials [36].

### Procedure

Subjects meeting full criteria on the online screen were contacted to participate in the experimental phase, and an appointment was made at that time. Upon arrival, each participant completed an Informed Consent Form. Subjects then completed the Medical History Questionnaire, CPDLT, CMHI, and STAXI. Consistent with previous research from this laboratory, only subjects scoring in the highest one-third of scores on the CMHI were considered to be experiencing high levels of cognitive anger [11, 27, 37, 38]. Scores over 29 on the CMHI were among the upper one-third of scores obtained by all participants in the online screening. Therefore, only those subjects with CMHI scores of 29 or higher were retained for the remainder of the experiment.

In order to compare anger expression styles, subjects were placed in groups based on STAXI scores on the anger in scale (AIS) and anger out scale (AOS). Cutoff scores for group inclusion were determined using clinical norms on the expression scales (AIS > 70th percentile = anger in; AOS > 70th percentile = anger out). Since some subjects did not cleanly fit into an expression category, due to both scales being elevated above the 70th percentile, individuals were grouped according to their highest elevation. For example, a subject scoring on the 73rd percentile on the anger in scale and the 95th percentile on the anger out scale would be placed in the anger out group. Assessment of anger expression style at the follow-up assessment revealed an unequal distribution of participants scoring higher on the A.I. (*n* = 33) and A.O. (*n* = 28) scales. A total of 17 participants demonstrated clinically significant elevations on both expression scales and were distributed between the A.I. (*n* = 12) and A.O. (*n* = 5) groups. As mentioned in the exclusion criteria, in order to ensure an equal distribution of individuals in each expression style group, a portion of participants who exhibited elevations on the A.I. scale were not retained for the experimental phase (*n* = 5). Final grouping resulted in an equal distribution of participants in both A.I. (*n* = 28) and A.O. (*n* = 28) groups, with a roughly equivalent distribution of individuals demonstrating significant elevations for either A.I. (*n* = 7) or A.O. (*n* = 5).

Baseline systolic blood pressure and heart rate data were collected from participants in accordance with previous procedures used in this laboratory [27, 30, 36], with two readings taken. A third reading was taken if the initial two readings were more than 10 mmHg (for systolic blood pressure) or beats per minute (for heart rate) apart. Following the baseline physiological measurements, participants completed measures of either verbal or figural fluency (Controlled Oral Word Association Test, COWAT [39]; Ruff Figural Fluency Test, RFFT [35]). Immediately following completion of the fluency measure, participants’ blood pressure and heart rate were taken as measures of physiological reactivity to the task. Following the first neuropsychological measure, participants completed the converse measure. Following completion of the second fluency test, blood pressure and heart rate data were obtained utilizing the method described above. Participants were given a 3-min rest period between the completion of the first task and the start of the second task. Neuropsychological test administration was counterbalanced to control for any possible order effects.

## Results

Anger expression groups did not differ in terms of laterality preferences (CPDLT; *t* = 1.53, 53, *p* = .879), reported tobacco smoking (Fagerstrom; *t* = −.537, 54, *p* = .593), CMHI scores (*t* = .769, 53, *p* = .445), baseline systolic blood pressure (*t* = .177, 54, *p* = .860), diastolic blood pressure (*t* = 1.122, 54, *p* = .267), and heart rate (*t* = .634, 53, *p* = .529). Mean values for baseline measures are presented in Table 1. An ANOVA was used for each trial, consisting of pre-task and post-task, comparing systole and heart rate consecutively, by anger expression style. Of note, some of the statistical power seen in the results may be from significant elevations in individual AI/AO scale scores versus an overall sample trend. However, to maintain the sample size and integrity of the overall data results, these scores were included in the statistical analysis. Bonferroni correction was used to correct for multiple comparisons.

**Table 1:** Baseline means and SD for CPDLT, fagerstrom, CMHI, systole, diastole, and heart rate

	Anger expression style	*N*	Mean	SD
CPDLT	In	28	9.39	1.59
	Out	28	9.32	1.88
Fagerstrom	In	28	.25	.52
	Out	28	.32	.48
CMHI	In	28	34.46	2.80
	Out	28	33.93	2.40
Systole	In	28	128.36	6.69
	Out	28	128.04	6.87
Diastole	In	28	70.00	3.78
	Out	28	68.79	4.30
Heart rate	In	28	73.57	2.73
	Out	28	73.11	2.75

### Systole

**Fig. 1:**
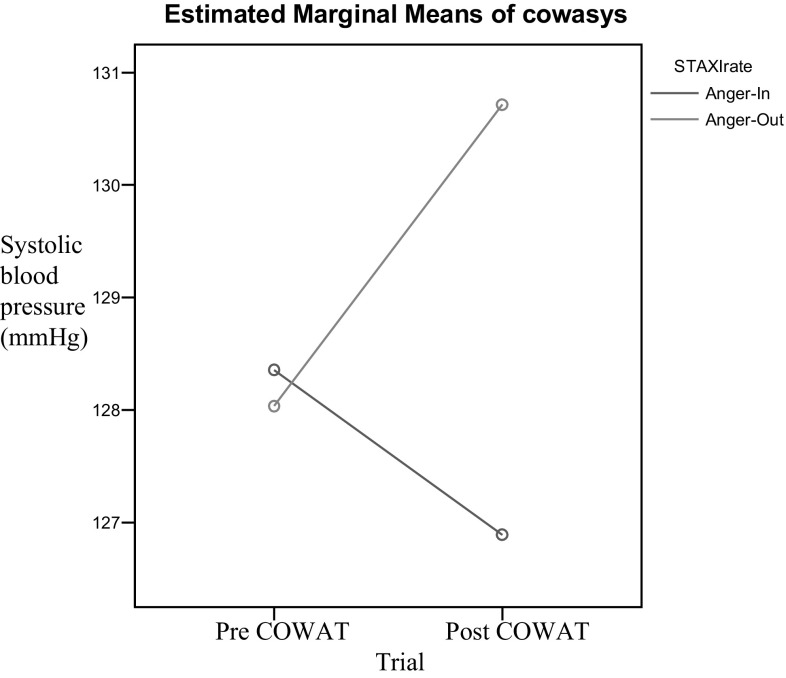
COWAT systolic blood pressure, group by time interaction

**Fig. 2:**
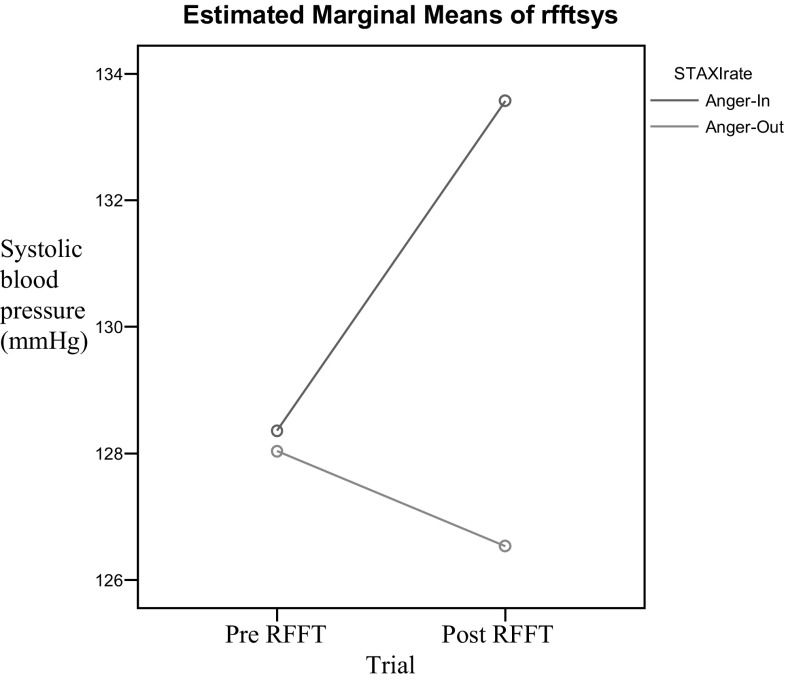
RFFT systolic blood pressure, group by time interaction

Consistent with the stated hypotheses, systolic blood pressure varied significantly as a function of anger expression style (Figs. 1, 2). Mean systolic blood pressure, diastolic blood pressure, and heart rate values after completion of the COWAT and RFFT are presented in Table 2. An ANOVA was used to examine the effects of verbal fluency (COWAT performance) on systolic blood pressure. No significant main effect of trial [*F*(1,54) = .94, *p* = .34] or anger expression style [*F*(91,54) = .937, *p* = .34] was found. However, there was a significant interaction of trial and anger expression style [*F*(1,54) = 10.89, *p* = .02]. The Cohen’s effect size value for the interaction (*d* = .508) indicated a moderate practical significance. Multiple comparisons of the interaction effects showed that participants in the A.O. expression style group demonstrated significantly higher systolic blood pressure following the completion of a verbal fluency measure [*t*(27) = −2.718, *p* = .−011]. However, our hypothesis that individuals in the A.O. group would demonstrate a significant decrease in systolic blood pressure following completion of the COWAT was not supported [*t*(27) = 1.88, *p* = .07]. Participants in the A.I. group did not show a significant decrease in systolic blood pressure after completing the verbal fluency measure.

**Table 2:** Means and SD for post-fluency systole, diastole, and heart rate

	Anger expression style	*N*	Mean	Standard deviation
COWAT systole	In	28	126.89	7.55
	Out	28	130.71	7.48
COWAT diastole	In	28	70.11	3.66
	Out	28	68.93	4.04
COWAT HR	In	28	72.61	4.19
	Out	28	73.39	3.20
RFFT systole	In	28	133.57	7.05
	Out	28	126.54	6.79
RFFT diastole	In	28	70.18	3.57
	Out	28	69.25	4.17
RFFT HR	In	28	73.29	4.19
	Out	28	72.79	3.20

In terms of the effects of a nonverbal fluency (RFFT performance) measure on systolic blood pressure, main effects were found for trial [*F*(1,54) = 16.67, *p* = .000] and anger expression style [*F*(1,54) = 4.30, *p* = .04], both in the hypothesized direction. Additionally, a significant trial by anger expression style interaction effect was found [*F*(1,54) = 54.47, *p* = .000]. The Cohen’s effect size for this interaction (*d* = 1.01) was high, suggesting that the observed difference was slightly greater than one standard deviation. Multiple comparisons of the interaction effect showed that participants in the anger in group demonstrated significant increases in systolic blood pressure [*t*(27) = 6.92, *p* = .000], and individuals in the anger out group demonstrated significant decreases in systolic blood pressure [*t*(27) = 4.10, *p* = .000].

### Heart rate

**Fig. 3:**
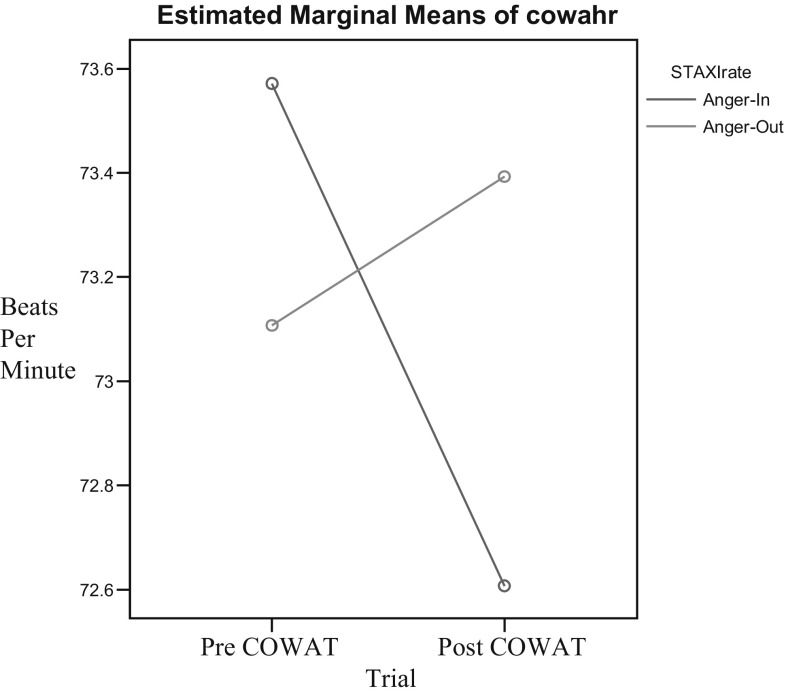
COWAT heart rate, group by time interaction

**Fig. 4:**
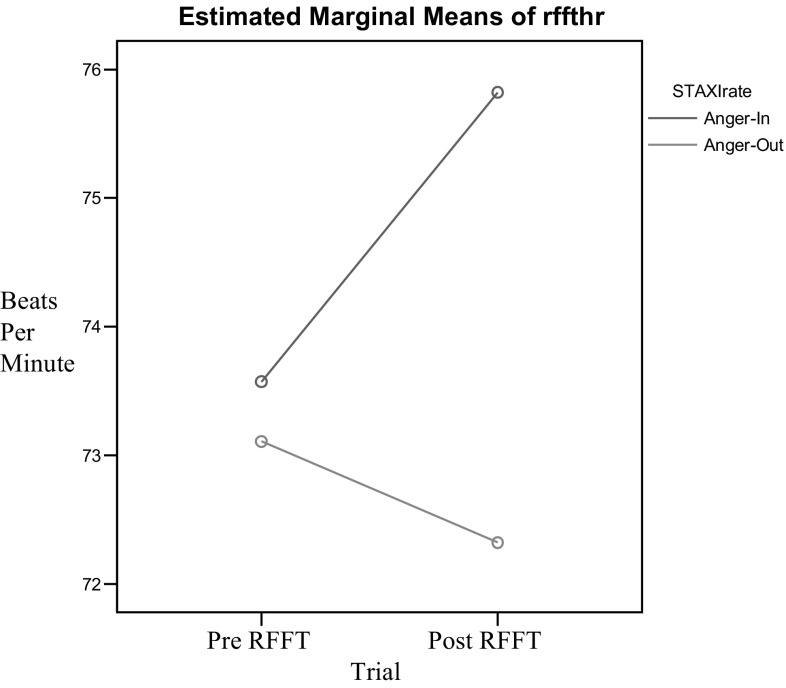
RFFT heart rate, group by time interaction

As predicted in the hypotheses, these results demonstrated significant changes in heart rate in response to completion of verbal fluency measure based on anger expression style (Figs. 3, 4). No significant main effect was found for trial [*F*(1,54) = 1.74, *p* = .193] or anger expression style [*F*(1,54) = .048, *p* = .83]. Conversely, there was a significant trial by anger expression style interaction effect [*F*(1,54) = 5.89, *p* = .02]. However, this interaction had a low Cohen’s effect size value (*d* = .229). Multiple comparisons of this interaction indicated that participants in the anger in expression style group demonstrated non-significant reductions in heart rate [*t*(27) = 1.09, *p* = .28]. Conversely, participants in the anger out group demonstrated a non-significant increase in heart rate following the completion of a verbal fluency measure [*t*(27) = 2.71, *p* = .03].

In terms of the effects of a nonverbal fluency measure on heart rate, main effects were found for trial [*F*(1,54) = 13.29, *p* = .001] and anger expression style [*F*(1,54) = 7.34, *p* = .009]. Again, these results were in the expected direction. Additionally, a significant trial by anger expression style interaction effect was found [*F*(1,54) = 57.12, *p* = .000]. The Cohen’s effect size value for this interaction (*d* = 1.27) was also high. Multiple comparisons of the interaction effect showed that participants in the anger in group demonstrated significant increases in heart rate [*t*(27) = −7.33, *p* = .000]. Conversely, individuals in the anger out group demonstrated significant decreases in systolic blood pressure [*t*(27) = 3.03, *p* = .005].

## Discussion

Hypotheses related to cardiovascular responses to lateralized neuropsychological stressors were generally supported by the current experiment. Significant interactions between anger expression styles and cardiovascular responses were found for both verbal and nonverbal fluency stressors. Participants reporting an expressive “anger out” (A.O) style demonstrated significant systolic blood pressure increases and a non-significant increase in heart rate following administration of a verbal fluency measure, while participants reporting a repressive “anger in” (A.I) style demonstrated non-significant decrease in these cardiovascular responses. Conversely, participants reporting a repressive A.I. expression style demonstrated significant increases in systolic blood pressure and heart rate following a measure of nonverbal fluency, while participants reporting an A.O. expression style demonstrated significant decreases in systolic blood pressure and heart rate to the same neurocognitive stressor.

These results are best explained under the framework of the quadrant model that, in conjunction with the theories of functional cerebral systems and cerebral space, forms the foundation for the anger expression model employed in this experiment. Among individuals reporting an expressive A.O. style, the model predicts greater utilization of left frontal resources, which is consistent with behavioral activation [13]. The addition of a verbal fluency stressor would provide further competition for left frontal resources, causing significant activation of the left frontal area. The concept of balance in the quadrant model predicts that significant activation of the left frontal region may lead to compensating deactivation of the right frontal region. As cardiac control has been linked to the right frontal region ([40], see also [13]), deactivation of the region would be expected to result in deregulation of cardiovascular responses. Conversely, among individuals reporting a repressive A.I. style, the left frontal activation produced by verbal fluency tasks is not competing for resources with the individuals’ anger expression style. Therefore, the frontal regions are balanced and the available right frontal resources allow for greater capacity to attend to cardiovascular regulation, resulting in a decrease in systolic blood pressure and heart rate.

Similarly, the cardiovascular responses to the nonverbal fluency measure among individuals reporting a repressive A.I. expression style are supportive of the quadrant model and the anger expression model proposed here. The anger expression model predicts greater utilization of right frontal resources (consistent with behavioral inhibition) for the repressive A.I. anger expression style. The additional stressor of a nonverbal fluency measure would provide competition for these frontal resources, resulting in increased right frontal activation. This right frontal activation is in direct competition for the right frontal resources, which have been demonstrated to be responsible for inhibition of right parietal and temporal regions. Increased activation of these temporoparietal regions have been proposed to be responsible for increases in sympathetic tone [41]. Therefore, significant increases in systolic blood pressure and heart rate in this condition are congruent with the predictions of the model.

The results regarding cardiovascular responses to the nonverbal fluency measure among participants reporting an expressive A.O. style are not readily interpreted under the framework of the quadrant model. One possible explanation for these results, congruent with the quadrant model, involves the element of balance. It may be that individuals with an expressive A.O. style have a greater capacity for design fluency tasks and therefore do not process this task as a cognitive stressor. Although there may be competition for right frontal resources by the design fluency task and cardiovascular control, the left frontal region is not activated (as the task is not stressful to this group, there is no activation of this region) and can balance the heightened activity of the right frontal region, allowing for greater control of cardiovascular functions.

Overall, the current experiment was supportive of the proposed anger expression theory within the larger context of the quadrant model. As suggested in the preceding literature review, the elements of competition and balance account for the bulk of the findings reported here. Direct competition for right frontal resources such as nonverbal fluency, muscular control of the left extremities, and regulation of systolic blood pressure demonstrated significant deregulation among participants reporting a repressive A.I. expression style. Direct competition may also be shown to cause individuals reporting an expressive A.O. style reduced efficiency in verbal fluency, as measured by perseverative errors. These results further suggest that hemispheric balance, as defined in the quadrant model, can be shown to cause deregulation of cardiovascular control and left-hand grip strength among individuals reporting an expressive A.O. style.

The current experiment was not without limitations. Future research in this area should be conducted utilizing a more diverse and representative sample. Although previous research does indicate that sex and handedness can impact lateralized functions, there is little research into the effects of education, age, and ethnicity on such tasks. In regard to sample heterogeneity, the findings here apply only to those individuals reporting higher levels of cognitive anger. In order to fully investigate the role that anger expression style may play in the findings of cerebral lateralization studies (e.g., [42, 43]), future experiments should be designed to examine these differences across levels of cognitive anger.

Another limitation for consideration is the usage of the identified neuropsychological instruments as indices of hemispheric activation. While the constructs used in the experiment are widely accepted as generally implicating left versus right activity, neither the RFFT nor the COWAT can be said to exclusively activate a given hemisphere. Finally, future investigations of anger need to provide greater focus into the role of the posterior cerebral systems. Although the results of this experiment demonstrate a clear relationship between frontal regions and anger expression styles, the potential contributions of posterior cerebral systems are not addressed. In particular, the potential role and neuroanatomical underpinnings of the right posterior region in relation to perception of anger and the left posterior region in relation to cognitive appraisals, and how these systems may be related to anger expression, warrant further investigation.

Given the association between anger and negative physical health outcomes (e.g., cardiovascular disease, increased risk of cerebrovascular accident, metabolic syndrome, and hypertension), there is a clear need to better understand the physiological components of this emotion. The results of this experiment indicate that a repressive A.I. expression style is associated with deregulation of the right frontal region. This same region has been shown to be intimately involved in cardiovascular recovery, glucose metabolism, and blood pressure regulation.
